# mHealth Interventions for Treatment Adherence and Outcomes of Care for Cardiometabolic Disease Among Adults Living With HIV: Systematic Review

**DOI:** 10.2196/20330

**Published:** 2021-06-09

**Authors:** Oluwakemi Ololade Odukoya, Chidumga Ohazurike, Maxwell Akanbi, Linda C O'Dwyer, Brenda Isikekpei, Ewemade Kuteyi, Idaomeh O Ameh, Olanlesi Osadiaye, Khadijat Adebayo, Adewunmi Usinoma, Ajoke Adewole, Nkiruka Odunukwe, Kola Okuyemi, Andre Pascal Kengne

**Affiliations:** 1 Department of Community Health and Primary Care College of Medicine University of Lagos Lagos Nigeria; 2 Department of Community Health Lagos University Teaching Hospital Lagos Nigeria; 3 Department of Preventive Medicine Feinberg School of Medicine Northwestern University Chicago, IL United States; 4 Galter Health Sciences Library and Learning Center Feinberg School of Medicine Northwestern University Chicago, IL United States; 5 Division of Nephrology Zenith Medical and Kidney Center Abuja Nigeria; 6 Department of Clinical Medicine All Saints University School of Medicine Roseau Dominica; 7 Non-Communicable Disease Research Group Nigeria Institute of Medical Research Lagos Nigeria; 8 Department of Family and Preventive Medicine University of Utah School Of Medicine Salt Lake City, UT United States; 9 Non-Communicable Disease Research Unit Medical Research Council Cape Town South Africa

**Keywords:** mHealth, HIV, cardiometabolic disease, text messaging, mobile, systematic review, telephone calls, wearable devices, smartphones, desktop, web-based, mobile apps

## Abstract

**Background:**

The success of antiretroviral therapy has led to an increase in life expectancy and an associated rise in the risk of cardiometabolic diseases (CMDs) among people living with HIV.

**Objective:**

Our aim was to conduct a systematic review to synthesize the existing literature on the patterns of use and effects of mobile health (mHealth) interventions for improving treatment adherence and outcomes of care for CMD among people living with HIV.

**Methods:**

A systematic search of multiple databases, including PubMed-MEDLINE, Embase, CINAHL, Scopus, Web of Science, African Journals online, ClinicalTrials.gov, and the World Health Organization Global Index Medicus of peer-reviewed articles, was conducted with no date or language restrictions. Unpublished reports on mHealth interventions for treatment adherence and outcomes of care for CMD among adults living with HIV were also included in this review. Studies were included if they had at least 1 component that used an mHealth intervention to address treatment adherence or 1 or more of the stated outcomes of care for CMD among people living with HIV.

**Results:**

Our search strategy yielded 1148 unique records. In total, 10 articles met the inclusion criteria and were included in this review. Of the 10 studies, only 4 had published results. The categories of mHealth interventions ranged from short messaging, telephone calls, and wearable devices to smartphone and desktop web-based mobile apps. Across the different categories of interventions, there were no clear patterns in terms of consistency in the use of a particular intervention, as most studies (9/10, 90%) assessed a combination of mHealth interventions. Short messaging and telephone calls were however the most common interventions. Half of the studies (5/10, 50%) reported on outcomes that were indirectly linked to CMD, and none of them provided reliable evidence for evaluating the effectiveness of mHealth interventions for treatment adherence and outcomes of care for CMD among people living with HIV.

**Conclusions:**

Due to the limited number of studies and the heterogeneity of interventions and outcome measures in the studies, no definitive conclusions could be drawn on the patterns of use and effects of mHealth interventions for treatment adherence and outcomes of care for CMD among people living with HIV. We therefore recommend that future trials should focus on standardized outcomes for CMD. We also suggest that future studies should consider having a longer follow-up period in order to determine the long-term effects of mHealth interventions on CMD outcomes for people living with HIV.

**Trial Registration:**

PROSPERO International Prospective Register of Systematic Reviews CRD42018086940; https://www.crd.york.ac.uk/prospero/display_record.php?ID=CRD42018086940

## Introduction

Cardiometabolic diseases (CMDs) represent a huge threat to the global progress that has been achieved in reducing mortality and morbidity among people living with HIV [1,2]. With the successes of antiretroviral therapies (ARTs), there has been increased life expectancy and a reduction in the burden of opportunistic infections among people living with HIV [3-5]. However, both HIV and ART are independently associated with an increased risk of CMD [6-8]. The incidence of CMD among people living with HIV ranges from 1.19 per 1000 person-years to 11.3 per 1000 person-years [9,10]. People living with HIV show consistent patterns of increased risk for diabetes [11-13], stroke [14], sudden cardiac death [15], heart failure [16], coronary heart disease, and myocardial Infarction [17-19]. Furthermore, established CMD risk factors like tobacco smoking and alcohol use are prevalent among people living with HIV [20,21].

The use of mobile technology, especially the use of mobile phones, has increased tremendously worldwide. The majority of the over 7 billion mobile phone users reside in low- and middle-income countries (LMICs), where the burden of HIV and AIDS is the heaviest [22]. The improved access to mobile technology presents an immense opportunity for promoting the health of people living with HIV, and mobile technology is increasingly being used to provide support for people living with HIV and promote adherence to ART [23-25].

Research on CMD among people living with HIV has increased significantly over the last 2 decades, and significant proportions of these studies have assessed the use of mobile health (mHealth) interventions to promote HIV care [7-12]. Systematic reviews on the topic have shown that most research in this field focuses on mobile phone interventions that aim to promote ART adherence and other forms of direct HIV and AIDS outcomes [25-30]. In a paper that systematically reviewed mobile phone SMS text messaging interventions for HIV and other chronic diseases, many of the studies with chronic disease outcomes did not include people living with HIV [30]. Thus, the evidence for analyzing the patterns of use and effects of mHealth interventions for CMD outcomes among people living with HIV remains unclear. This review aims to synthesize existing literature on the patterns of use and effects of mHealth interventions for treatment adherence and outcomes of care for CMD among adults living with HIV.

## Methods

### Study Design

For this review, we followed the guidelines from the PRISMA (Preferred Reporting Items for Systematic reviews and Meta-Analyses) Statement [31]. The study protocol was also registered with the International Prospective Register of Systematic Reviews (trial ID number: CRD42018086940). We relied on data obtained from other studies that conducted primary research; therefore, obtaining institutional ethical approval was not required. We made use of deidentified data that were stored in a password-protected database. We ensured that the data presented in this systematic review did not violate the privacy of the patients.

### Eligibility Criteria

#### Inclusion Criteria

To ensure that we captured all articles that described patterns of existing mHealth interventions for CMD outcomes, we did not limit our search by study type or design. We included studies that described any mHealth interventions if the following criteria were met: (1) the study was conducted among adults aged ≥18 years living with HIV; (2) the study involved the use of mobile phones or had any mHealth components embedded in its design; and (3) the study was designed to influence adherence to treatment or outcomes of care for 1 or more CMD (ie, hypertension, dyslipidemia, obesity, stroke, coronary heart disease, diabetes mellitus, and metabolic syndrome). Articles published in any language with English abstracts were eligible for inclusion.

#### Exclusion Criteria

A study was excluded if (1) it was an opinion piece, (2) it was a publication that lacked primary data, and (3) it had no explicit method description. In the case of duplicate publications of the same material in more than 1 journal or conference proceeding, the most complete and recent version was used.

#### Definitions

The diagnosis of HIV was based on positive reports from screening tests or participants’ self-reported doctor diagnoses. The CMDs reviewed included hypertension, diabetes mellitus, dyslipidemia, obesity, stroke, coronary heart disease, and metabolic syndrome. This study was a systematic review for which no primary data were collected. The diagnosis of HIV and cardiometabolic outcomes were based on reports from the studies included in this review.

The outcome measures included differences in adherence to treatment or outcomes relating to any of the listed CMDs; differences in mortality due to the listed CMDs; differences in blood pressure, glycemic control, and blood lipid levels; and reductions in CMD risk or BMI waist circumference and waist-hip ratios.

With regard to mHealth, for the purpose of this review, we used the Global Observatory for eHealth definition, which defines mHealth as a medical and public health practice supported by mobile devices, such as mobile phones, patient monitoring devices, PDAs, and other wireless devices. mHealth involves the use and capitalization of a mobile phone’s core utility in terms of voice messaging services and SMSs as well as more complex functionalities and applications, including general packet radio services, third and fourth generation mobile telecommunications (3G and 4G systems), GPSs, and Bluetooth technology [32].

### Search Strategy for the Identification of Relevant Studies

Electronic searches of the following databases were conducted from inception to September 2019: PubMed-MEDLINE, Embase, CINAHL, Scopus, Web of Science, Cochrane Central Register of Controlled Trials, Global Health (Elton B. Stephens Company), the Institute of Electrical and Electronics Engineers, African Journals online, the Association for Computing Machinery, World Health Organization (WHO) reports, ClinicalTrials.gov, The Pan African Clinical Trials Registry and mHealth alliance, and the WHO Global Index Medicus. The search terms used included HIV-related terms (eg, *HIV infections*, *HIV*, *HIV/AIDS*, *HIV-positive*, and *HIV infected*), mobile device–related terms (eg, *mobile health*, *mHealth*, *mobile phone*, and *short message*) and CMD-related terms (eg, *Hypertension*, *Diabetes*, *stroke*, *metabolic syndrome*, and *cardiometabolic*). No language, publication type, or date limits were applied to the initial searches. The first author (OOO) and the librarian (LO) collaboratively developed the search strategy. The full list of search strategies is available in Multimedia Appendix 1.

### Reference Lists and Grey Literature

We searched for additional relevant articles in the reference lists of the retrieved key articles and reviews. We contacted authors of included studies to acquire other data related to our outcomes of interest that may have been unpublished, informally published, or undergoing ongoing analysis. Principal investigators of registered clinical trials whose published results were not found within our search were contacted by email to share their results.

### Data Collection and Processing

**Figure 1:**
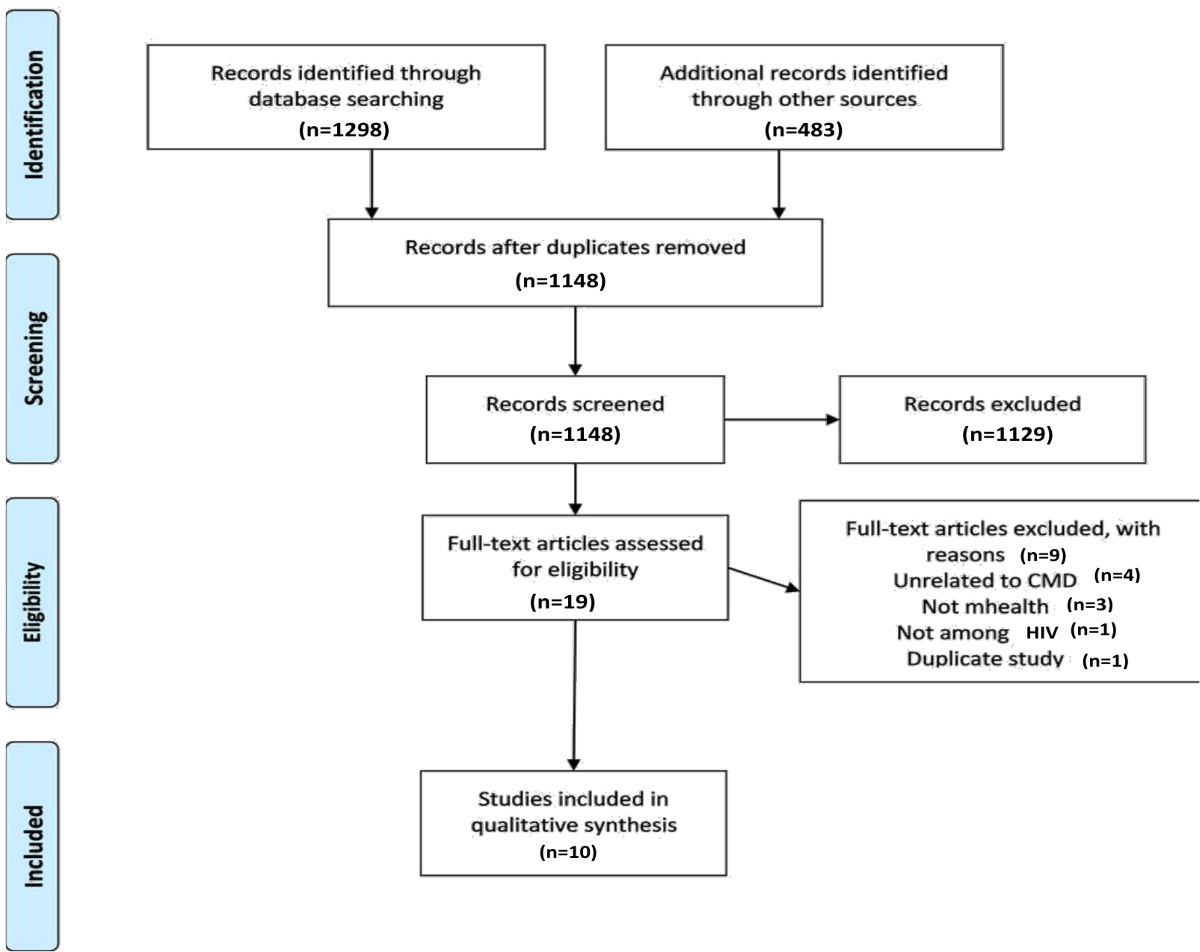
PRISMA (Preferred Reporting Items for Systematic Reviews and Meta-Analyses) flowchart. CMD: cardiometabolic disease.

Search results were saved into EndNote (Clarivate Analytics) files by the librarian (LO). All EndNote files were deduplicated, collated, and transferred into Rayyan (Rayyan Systems Incorporated) [33] for subsequent processing. A pilot screen of 100 articles was performed by each reviewer to ensure the consistent interpretation of the inclusion and exclusion criteria. Two sets of reviewers (set 1: BI and KA; set 2: OO and OU) conducted an initial independent screening and eligibility assessment of articles’ titles and abstracts by using the predefined inclusion and exclusion criteria. A third reviewer (OOO) resolved disagreements. Full-text copies of the selected articles were obtained for further review and assessed using the same process as the title and abstract screens. The flowchart for the study selection process is shown in Figure 1. Two independent reviewers (BI and OO) used a pretested data extraction form that was adapted from the Cochrane data extraction template for intervention reviews of randomized controlled trials (RCTs) and non-RCTs [34] (Multimedia Appendix 2) to extract the data from the full texts [35-38]. The information extracted included the study title, author, year, country, study design, sample size, study population and setting, intervention type and delivery, components of the intervention, concurrent non-mHealth interventions or medications (if any), duration of intervention, cardiometabolic outcome, and secondary outcome measures. The results were synthesized and presented as a narrative synthesis of the details regarding the study type, intervention characteristics, study outcomes, and location. Due to the limited number of studies and the wide variability in the outcome measures, we were unable to perform a meta-analysis.

### Methodological Quality of Included Studies

The quality of included studies was assessed by using the Cochrane risk of bias assessment tool [39]. Specifically, we assessed the quality of each included study by using the criteria from the seven domains of the tool (ie, random sequence generation, the blinding of study participants and key personnel, the blinding of the outcome assessment, selective outcome reporting, allocation concealment, incomplete outcome data, and the presence of bias from other sources) [39]. For each of these domains, each study was identified as having a low, high, or unclear risk. In total, 2 studies did not report on the blinding of the outcome assessment [35,36], while 3 studies had incomplete outcome data [35,36,38]. Two investigators (BI and OO) independently assessed the quality of included studies, and a third reviewer (OOO) resolved discrepancies.

## Results

### Included Studies

Our search yielded 1148 unique records, of which 1129 were excluded for the reasons specified in Figure 1. Most of the excluded records (1129/1148, 98.34) were not related to mHealth, not related to our CMD outcomes of interest, or not conducted among people living with HIV. Of the 19 articles that were fully reviewed, 4 were unrelated to the CMD outcomes of interest, 3 were not about mHealth interventions, 1 was not conducted among people living with HIV, and 1 was a duplicate study. In total, 10 articles met the inclusion criteria.

The 10 included studies and their key study characteristics are summarized in Table 1. Of these included studies, 7 were RCTs [35,36,40-43]. In 90% (9/10) of studies, study participants only included people living with HIV. The remaining 10% of studies included a combination of both people living with HIV and people living without HIV. Most studies (4/10, 40%) had an average study period of about 12 months [36,37,40,43]. It is noteworthy that 6 of the 10 included studies were registered clinical trials that had unpublished results at the time of conducting this review [40-45]. The principal investigators of the included registered clinicals trials were contacted in order to ascertain the progress of the trials; however, no response was received at the time of concluding this review. The number of studies with unpublished results and the variation in outcomes across studies made it difficult to conduct a meta-analysis.

**Table 1:** Summary of the key characteristics of included studies (N=10).

Study (author, year, country)	Study design and methods	Inclusion criteria	Interventions	Outcomes	Reported results
Morillo-Verdugo et al [36], 2018, Spain	Randomized controlled trial	Aged >35 years; on antiretroviral therapy with at least 1 drug prescribed for the treatment of hypertension, dyslipidemia, angina pectoris, cardiovascular prophylaxis, or type 2 diabetes mellitus; and at a moderate or high risk of cardiovascular disease	Periodic text messages on mobile phones	Cardiovascular risk index, smoking reduction, blood pressure control, and medication adherence	20.7% of patients in the intervention group vs 12.5% of patients in the control group reduced their Framingham risk score from high/very high to moderate/low (*P*=.02), and the number of patients with controlled blood pressure increased by 32.1% (*P*=.01). 37.9% of patients overall stopped smoking (*P*=.001).
Anglada-Martinez et al [35], 2016, Spain	Single-arm, prospective pre- and postintervention study	Patients on treatment for heart failure, hypertension, or dyslipidemia for >1 month and those aged >18 years	Medplan smartphone app and weekly motivational messages	Medication adherence, cholesterol, triglycerides, and blood pressure control	The proportion of missed doses decreased significantly for patients using the Medplan app (*P*<.05). There was no difference in the health outcomes of patients.
Roos et al [37], 2014, South Africa	Randomized controlled trial	On antiretroviral therapy for >6 months, aged 20-65 years, ambulatory without assistive device, and had an elevated risk of ischemic heart disease	Pedometer, activity diary that included education materials and documents for self-monitoring, and 1 monthly cell phone SMS message for motivation	The pedometer step count of both groups improved significantly.	The pedometer step counts of the control and intervention groups improved significantly (*P*=.03 for both groups) at 6 months, but this improvement was not significant at 12 months (*P*=.33 and *P*=.21, respectively). Significant between-group effects were observed in 6-minute walk test distances (*P*=.01), waist-to-hip ratios (*P*<.01), glucose levels (*P*<.01), and high-density lipoprotein levels (*P*<.01) over the 12-month period.
Zuniga et al [38], 2019, United States of America	1-group pre- and posttest design	Aged >18 years and had HIV and type 2 diabetes mellitus	6-hour educational instruction implemented as 2 3-hour meetings followed byweekly telephone support calls for 6 weeks	Diabetes self-management skills and knowledge about HIV or diabetes	There was a 34% increase in diabetes self-management skills from pretest to posttest, but there were no changes in knowledge about HIV or diabetes.
Grinspoon [40], 2006, United States of America	Randomized case control study	Aged 18 to 65 years and had 3 of the following 5 characteristics: (1) waist circumferences of >102 cm (40 in) for men and >88 cm (35 in) in women; (2) triglyceride levels of ≥150 mg/dL or current antilipolytic drug treatment; (3) high-density lipoprotein levels of <40 mg/dL for men and <50 mg/dL for women; (4) blood pressure of ≥130/85 mmHg or current antihypertensive drug treatment; and (5) fasting glucose level of ≥110 mg/dL	1-time counseling session with nutrition staff at the baseline visit and monthly unscripted phone calls	Waist-hip ratios and cardiovascular indices (total cholesterol; low-density lipoprotein, high-density lipoprotein, and triglyceride cholesterol levels; blood pressure; cardiac enzymes; C-reactive protein; tissue plasminogen activator; plasminogen activator inhibitor; insulin; and glucose metabolism)	The results of the study have yet to be published.
Jaggers et al [41], 2013, United States of America	Randomized controlled trial	Aged >18 years, had a sedentary lifestyle, had a viral load of >75 copies/mL, was capable of performing required exercise regimen, and had daily access to a telephone for approximately 10 months	Home-based physical activity intervention: The intervention included a 60-min, individual, face-to-face session; telephone counselling calls; and educational workbooks and pedometers for the self-monitoring of physical activity.	The effect of the intervention in terms of decreasing modifiable risk factors and increasing physical activity among people living with HIV and the effect of the intervention in terms of decreasing modifiable risk factors, such as fat distribution, blood lipids, and cardiorespiratory fitness outcomes, were assessed.	The findings of the study have yet to be published.
Brooke [42], 2017, United States of America	Randomized controlled trial	People living with HIV	Personalized, automated, interactive mobile phone text message intervention	Physical activity and dietary assessments; polyunsaturated fatty acids, carotenoids, and other biomarkers in plasma; and total cholesterol, triglyceride, and high- and low-density cholesterol	The study is still ongoing.
Dodson et al [43], 2016, Australia	Cluster randomized controlled trial	Aged >30 years, was receiving care from a participating doctor, was not diagnosed with cardiovascular disease, and had not participated previously in an HIV-specific self- management or coaching program	Health map website for (1) routine clinic visits involving the sharing of health records with a doctor; (2) access to own health record and information from home; (3) access to telephone and web-based self-management support; and (4) access to a peer-moderated, web-based group chat program.	10-year risk of nonfatal acute myocardial infarction or coronary heart disease death, as estimated by a Framingham Heart Study risk equation and the Positive and Active Engagement in Life Scale from the Health Education Impact Questionnaire	The findings of the study have yet to be published.
Oduor et al [44], 2018, Kenya	Contextual user interviews	Patients living with HIV and hypertension	Integrated desktop and mobile app	Improved efficacy, safety, and personalization of medication prescription	Descriptive study
Kengne [45], 2019, South Africa	Randomized controlled trial	Adult South Africans with comorbid HIV and hypertension	Automated text messaging	Mean difference in systolic and diastolic blood pressure at baseline and follow-up, uptake and adherence to blood pressure medications, mean change in lipid variables, and mean change in adiposity variables	The results of this study have yet to be published.

All included studies had an mHealth component, although some studies used a multimethod mHealth intervention approach, wherein support telephone calls were combined with educational instructions [38,41] or the use of mobile phone apps were combined with user interviews [44]. However, short messaging [36,37,42,45] and telephone calls [38,40,41,43] were the most common mHealth interventions. Furthermore, 2 studies used mobile apps as their mHealth intervention component [35,44].

Although studies had varied outcomes, most had treatment adherence (1/10, 10%) [44], cardiometabolic outcomes (5/10, 50%) [37,40-43], or both (3/10, 30%) [35,36,45] as their primary or secondary study outcomes. The other studies had a combination of other outcomes that were not limited to treatment adherence or cardiometabolic outcomes, such as physical activity and the self-management of diabetes [38,42].

With regard to the function of mHealth interventions, 4 studies used mHealth for medication adherence purposes [35,36,44,45], 3 studies used mHealth to improve physical activity and thereby reduce cardiovascular risk [37,41,42], 2 used mHealth for health promotion and health coaching purposes to reduce cardiovascular risk [37,38], and 1 used mHealth for the self-management of diabetes mellitus [38].

The majority of the studies (9/10, 90%) were conducted in hospital or clinic settings [35-38,40-43,45], whereas 10% (1/10) of the studies were conducted in a community setting in a rural area [44]. Furthermore, most of the studies were conducted in the high-income countries of Spain (2/10, 20%), the United States of America (4/10, 40%), and Australia (1/10, 10%). Only 3 studies were conducted in the LMICs of South Africa and Kenya in Sub-Saharan Africa, where the burden of HIV is quite significant.

### Effectiveness of mHealth Interventions

With regard to the effects of mHealth interventions on treatment adherence and cardiometabolic outcomes in the 4 studies with published results, 2 studies reported a decrease in cardiovascular risk [36,37] and significant group effects for cardiometabolic outcomes, such as those on glucose level and high-density lipoproteins, between intervention and control groups [37]. In total, 1 study reported a 34% increase in diabetes self-management skills among participants [38], and 1 study reported no differences in effects on treatment adherence among study participants [35].

### Assessing the Risk Of Bias

**Figure 2:**
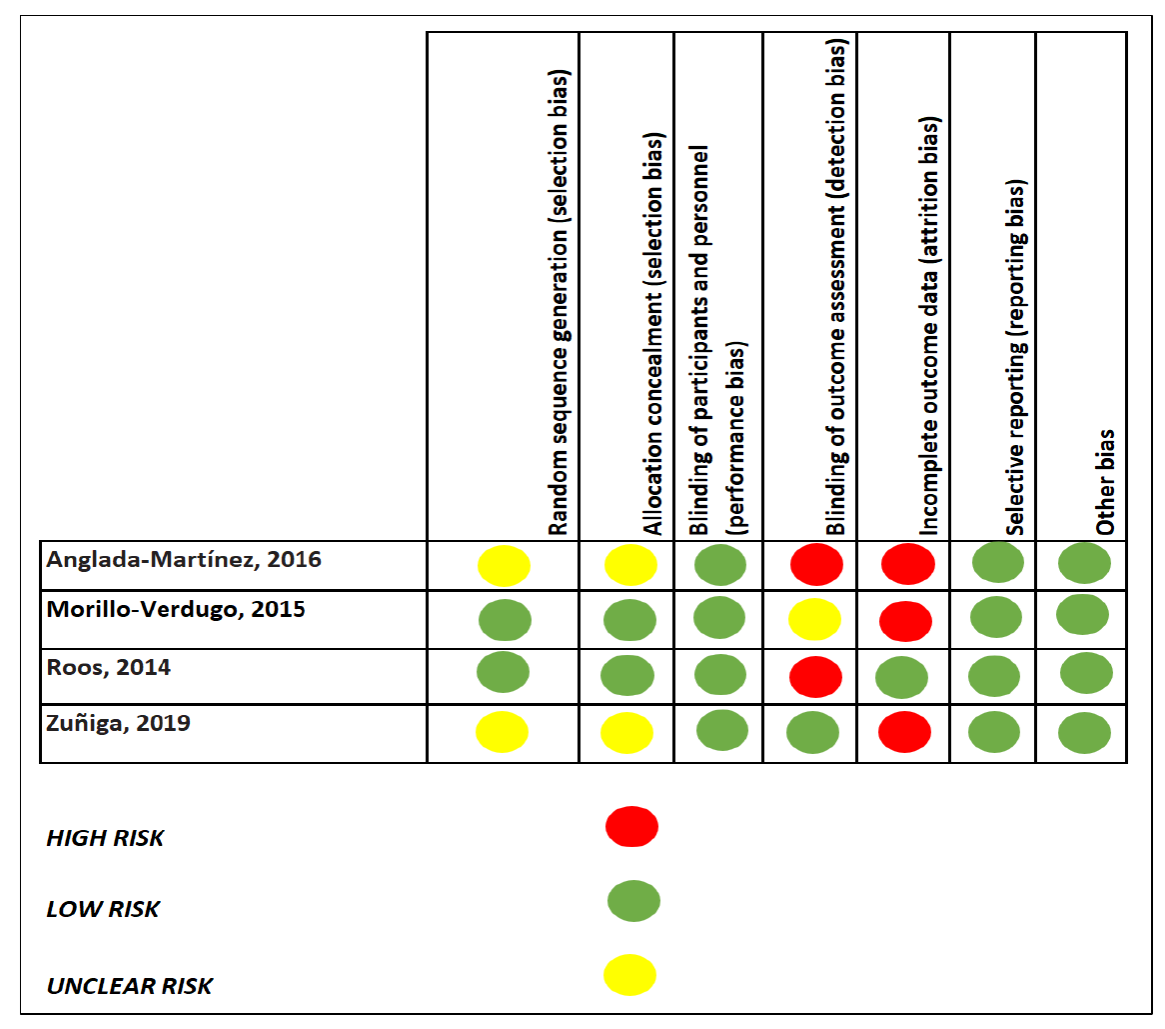
Cochrane risk of bias assessment [35-38].

Figure 2 shows the assessment of the risk of bias in the included studies.

#### Selection Bias (Random Sequence Generation and Allocation Concealment)

Among the 10 included studies, the assessment of bias was conducted in only the 4 studies that were completed (Anglada-Martinez et al [35], Morillo-Verduogo et al [36], Roos et al [37], and Zuniga et al [38]). In the study by Morillo-Verduogo et al [36], the participants were randomized into control and intervention groups with a specific software that was used to generate a sequence of random numbers. Roos et al [37] used a simple randomization formula in Microsoft Excel 2010 for randomization. The method for selecting participants was unclear in the study by Anglada-Martinez et al [35], as the patients were followed during the preintervention phase in accordance with their usual schedule. Zuniga et al [38] selected 25 participants by using a convenient sampling method. This was because the Zuniga et al [38] study had to be completed in 12 months due to the guidelines of their funding mechanism as well as the constraints from budget limitations. During the allocation of participants, Roos et al [37] used an academic who was not directly involved in the study to carry out allocation concealment via sequentially numbered envelopes. In the studies by Zuniga et al [38] and Anglada-Martinez et al [35], allocation concealment was not carried out for participants. This was because the Zuniga et al [38] study was a single-group study with a pre- and posttest study design, and in the Anglada-Martinez et al [35] study, the sequence generation was not described.

#### Performance and Detection Bias (Blinding of Participants, Personnel, and the Outcome Assessment)

Only 1 out of the 4 completed studies carried out blinding, and this was the blinding of the study personnel. In that study the research assistant performed all of the assessments and was blinded to the group allocation. The assessment forms were also coded to ensure anonymity, and the first author conducted the intervention [37].

The studies by Anglada-Martinez et al [35] and Morillo-Verduogo et al [38] did not record the blinding of participants and personnel; however, this was unlikely to influence the outcomes of those studies. The blinding of participants and personnel was not conducted in the Zuniga et al [38] study, as the graduate research assistant already knew the participants because they recruited, enrolled, and collected the baseline and follow-up data. This may likely have had an influence on the outcome of the study.

With regard to detection bias, the study by Anglada-Martinez et al [35] noted that the participants were not blinded to the measurement of the outcomes. This may have resulted in a Hawthorne effect, whereby the participants may have modified their behaviors because they were aware that their adherence to medication was being observed, thereby influencing the outcome of the study. Although the assessment forms were noted to be coded in the study by Roos et al [37], the blinding of the participants in the intervention group was not performed. These participants had 5 contact sessions in which they were educated on ischemic heart disease risk factors and the benefits of and methods for increasing physical activity levels. The participants in the control group experienced an increase in their pedometer step counts, which may have been linked to the fact that they were aware of their behavior (the Hawthorne effect). These factors were likely to influence the outcome of the Roos et al [37] study. Additionally, during the completion of the study, the intervention participants reported that wearing the pedometer motivated them to increase their activity levels [38]. The studies by Morillo-Verduogo et al [36] and Zuniga et al [38] failed to describe whether any form of blinding for the outcome assessor was performed.

#### Attrition and Reporting Bias (Incomplete Outcome Data and Selective Reporting)

In the study by Anglada-Martinez et al [35], there was a loss to follow-up of 20 participants. They were lost as a result of changing their addresses (n=3), transitioning to the use of a non-Android or non-iOS device (n=2), having incompatible Android and iOS operating systems (n=6), dying (n=1), not attending follow-ups (n=7), and withdrawing from the study (n=1). It was also noted that the adherence rates recorded with the use of the app did not correlate with the adherence rates that were recorded by analyzing the proportion of days in which participants took their medication. Some participants experienced problems such as their reminders not working properly (50%), and some believed that the use of the app resulted in extra work (fourth month: 60.2%; this decreased to 56.6% in the sixth month). Further, pending Medplan alerts, which appear during the day, disappear at midnight; hence, patients who took their medication before midnight were unable to confirm that they had done so after midnight (57.1%) [35]. The prespecified outcomes of interest were however reported in accordance with the study protocols [35].

In the study by Morillo-Verdugo et al [36], there was loss to follow-up of 5 participants, and 1 died from causes that were beyond the scope of the study. It was also noted that because of the low number of patients with diabetes in the study, glycosylated hemoglobin was not included in the analysis. The prespecified outcomes of interest, such as reductions in cardiovascular risk indices based on Framingham scores, adherence to ART, and lifestyle modifications, were reported in accordance with the study protocol [36].

In the study by Roos et al [37], there were losses to follow-up in the control group at 6 months (n=8) and 12 months (n=10) and in the intervention group at 6 months (n=3) and 12 months (n=6). In the control group, 3 participants did not return for their second baseline assessment, and their baseline blood results were managed by imputing the mean value of the cohort’s results. Additionally, some data were noted to be missing completely at random, and these were managed by imputing the last observation that was carried forward [37]. The prespecified outcomes of interest, such as perceived stress, physiological measures, physical activity, physical function capacity, biochemical measures, and Framingham risk scores, were reported in accordance with the study protocol [37].

In the study by Zuniga et al [38], 15 out of 25 patients participated in the baseline fasting blood tests, but only 1 completed the blood drawing for the collection of follow-up data. Therefore, the effects of the intervention on HIV or diabetes control could not be recorded. The reasons for losses to follow-up were not described; however, the reporting of prespecified outcomes of interest, such as the knowledge of diabetes and HIV and diabetes self-management activities, were reported in accordance with the study protocol [38].

## Discussion

### Principal Findings

This paper presents a narrative synthesis of mHealth interventions for treatment adherence and outcomes of care for CMD among adults with HIV. A total of 10 studies met the inclusion criteria and were included in the review. The majority of studies included in this review were conducted in high-income countries (7/10, 70%) [35,36,38,40-43], and only a handful of studies were from LMICs (3/10, 30%) [37,44,45].

Based on our review, the categories of the interventions that were used in the studies ranged from short messaging and telephone calls to wearable devices and smartphone and desktop web-based mobile apps. However, the two most common interventions that were provided to people living with HIV were short messaging and telephone calls. This finding is similar to those from another systematic review on the impact of mHealth chronic disease management on treatment adherence and patient outcomes, which found that 40% of the studies included in their review had used short messaging to track medication adherence in patients with chronic diseases [46]. We also found that across the different categories of interventions, there were no clear patterns in terms of consistency in the use of a particular intervention, as most studies (9/10, 90%) [35,36,38,40-45] assessed a combination of mHealth interventions.

Overall, authors reported that the use of mHealth interventions for treatment adherence and outcomes of care for CMD among adults living with HIV was effective. However, studies varied widely in terms of research questions, target groups, study outcomes, and settings [36,37,41,43,44]. The risk of bias varied from study to study. Of the 4 studies that assessed the risk of bias, 2 controlled for selection bias by randomization [35,37], and only 1 study performed the blinding of both participants and research personnel for the control of performance bias [37]. All 4 studies reported their prespecified outcomes of interest in accordance with their study protocols.

Most existing studies on mHealth interventions for people living with HIV have addressed ART adherence outcomes, and only a few have assessed mHealth interventions for CMD outcomes. In theory, the use of mHealth interventions to monitor treatment adherence and outcomes of care for both ART and CMD should make the process of care more efficient. However, research in this area is still very limited. This highlights the need to generate evidence to promote the use of integrated models of care for outcomes such as ART adherence and CMD outcomes. In addition, our findings from this review revealed that most studies did not report outcomes such as a reduction in the incidence of CMD, which should be the ultimate goal, given the increasing life expectancy of people living with HIV resulting from ART. However, this could be explained by the short follow-up periods that were used in these studies. We highlight the existing mHealth interventions that specifically target CMD outcomes among people living with HIV and draw attention to the gaps and opportunities in mHealth interventions for comorbid CMDs among people living with HIV. Furthermore, our review shows the paucity of well-designed RCTs in this research area. We also call attention to the disparities in the conduct of research on this topic. Globally, the WHO African region has been and remains to be the most severely affected by the HIV epidemic, as it accounts for more than two-thirds of the people living with HIV worldwide and nearly 3.7% adults (about 1 in every 25) living with HIV [47]. However, only 1 out of the 4 completed studies and 3 out of the 10 reviewed studies were conducted in an LMIC setting.

Although we purposefully used broad inclusion criteria to capture all studies evaluating any type of mHealth intervention for CMD among people living with HIV, we were limited by the low number of studies that met our inclusion criteria or reported on our predefined key outcomes. Furthermore, many of the studies that fit our inclusion criteria were old. Therefore, they may not have reflected the current state of the effectiveness of mHealth interventions. We also could not conduct a meta-analysis due to the heterogeneity of the included studies in terms of their methods and reported outcomes. However, it is important to point out that a number of clinical trials are underway, and their results can be incorporated in a follow-up review within the next few years. We recommend that future trials should focus on standardized outcomes for CMD to enable the conduction of a meta-analysis. We also suggest that future studies should consider using an integrated approach and a longer follow-up period in order to determine the long-term effects of mHealth interventions on outcomes of care.

### Conclusion

Studies using mHealth interventions that specifically target CMD outcomes for people living with HIV are limited, particularly in Sub-Saharan Africa, where the burden of HIV is the greatest. In this review, although several of the mHealth interventions were found to be effective, there appears to be no clear pattern in the use of mHealth interventions for CMD outcomes. Short messaging was the most used intervention. More studies that assess the use and effectiveness of mHealth interventions other than short messaging, such as mobile apps and wearable health devices, are needed in this study area.

## Appendix

Multimedia Appendix 1 Search strings.

Multimedia Appendix 2 Data extraction template [<xref ref-type="bibr" rid="ref35">35</xref>-<xref ref-type="bibr" rid="ref38">38</xref>].

## Abbreviations

ART: antiretroviral therapy
CMD: cardiometabolic disease
LMIC: low- and middle-income country
mHealth: mobile health
PRISMA: Preferred Reporting Items for Systematic reviews and Meta-Analyses
RCT: randomized controlled trial
WHO: World Health Organization
