# Supplementary figures and images for: mHealth Interventions for Treatment Adherence and Outcomes of Care for Cardiometabolic Disease Among Adults Living With HIV: Systematic Review
Source: JMIR Mhealth Uhealth. 2021 Jun 9;9(6):e20330. doi: 10.2196/20330 (PMC8409010; doi:10.2196/20330)

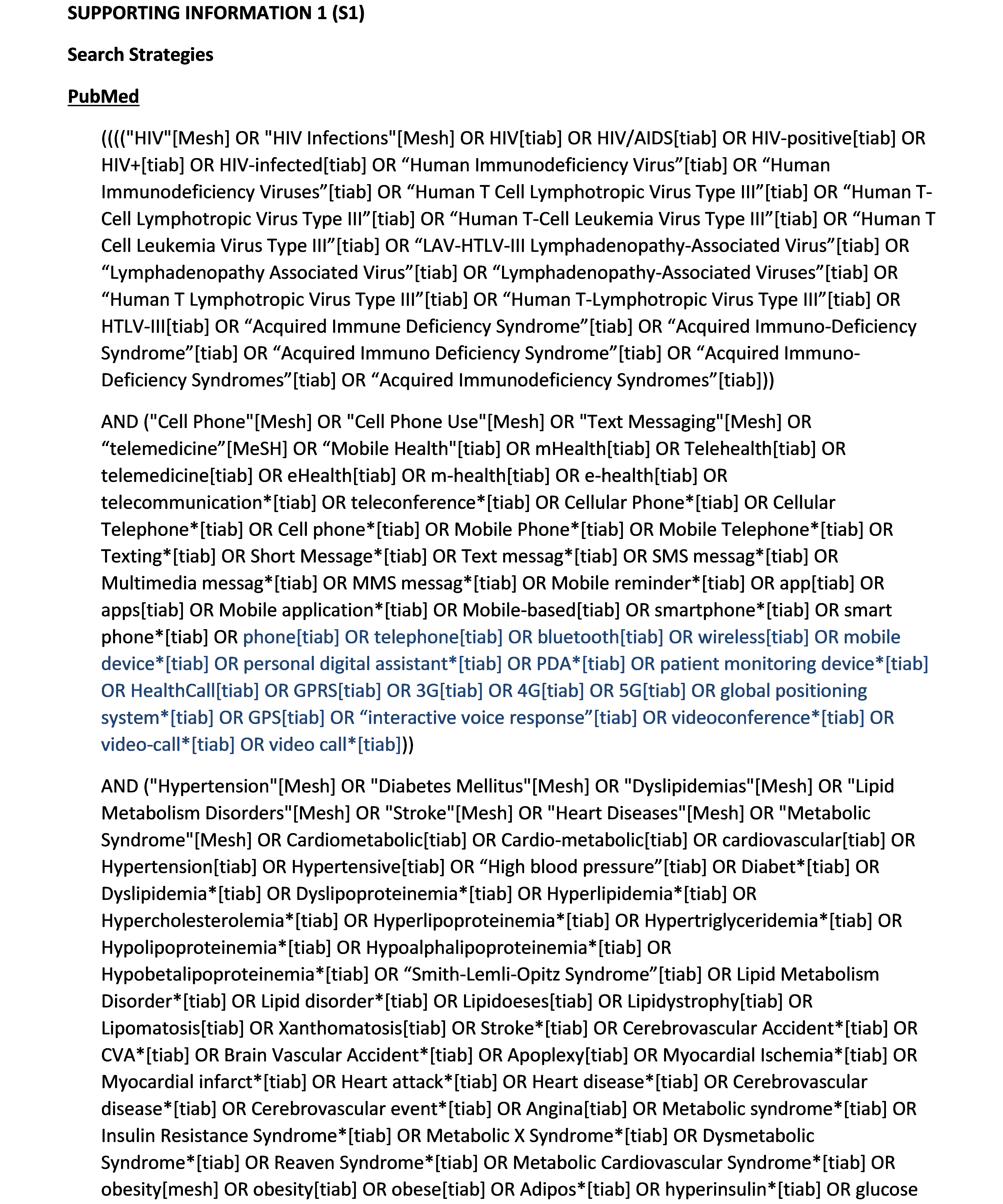

Supplement: Multimedia Appendix 1 [file mhealth_v9i6e20330_app1.png]

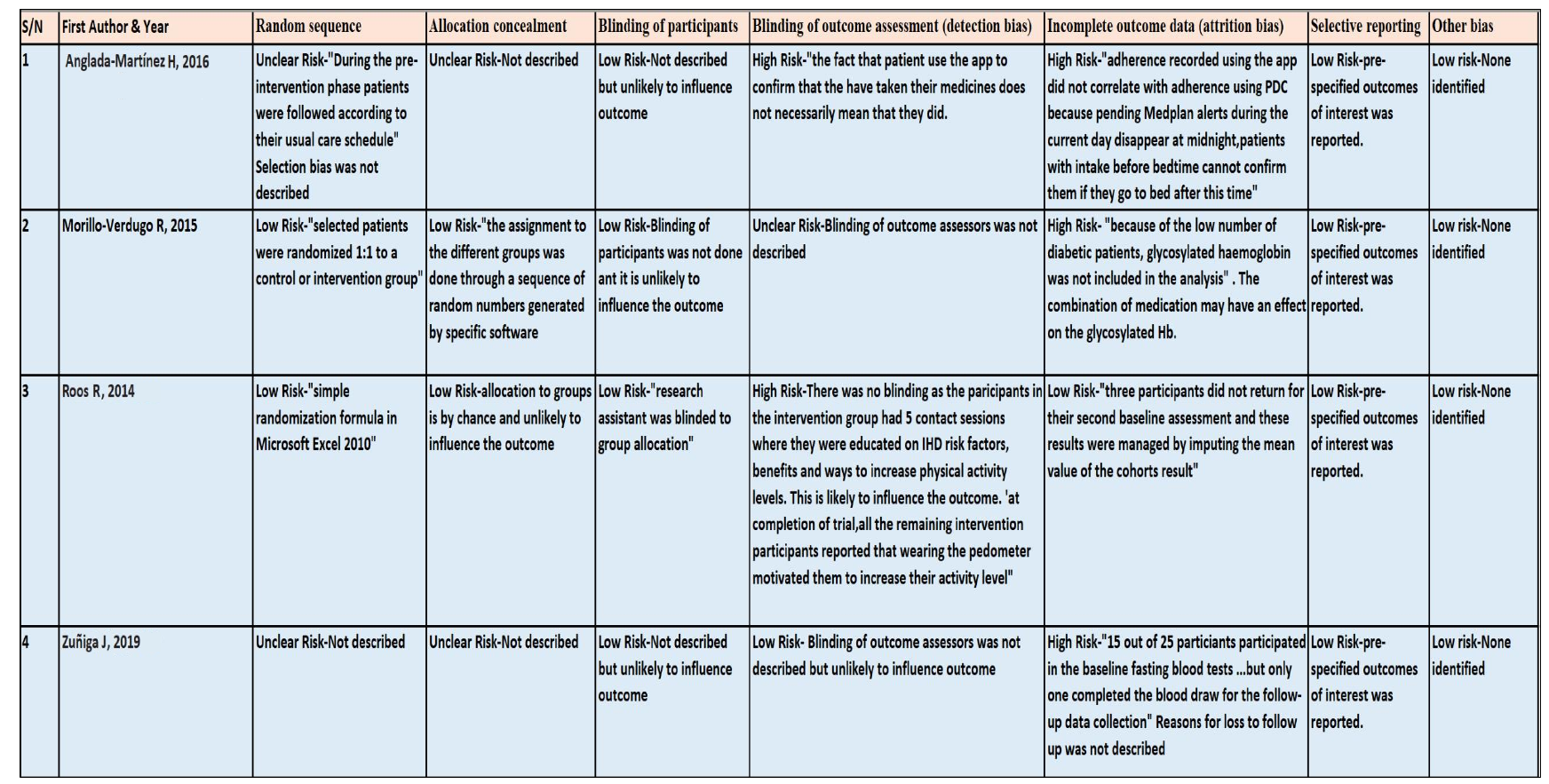

Supplement: Multimedia Appendix 2 [file mhealth_v9i6e20330_app2.png]
